# Challenges in treating people with Parkinson’s disease during the COVID-19 pandemic

**DOI:** 10.1186/s43161-020-00017-5

**Published:** 2020-11-25

**Authors:** Thiago da Silva Rocha Paz, André Ricardo Silva de Macedo, Ana Elisa Lemos Silva, Girlene Pessoa, Núbia Isabela Macedo Martins, Humberto Lameira Miranda, Vera Lúcia Santos de Britto, Clynton Lourenço Corrêa

**Affiliations:** 1 Graduate Program in Physical Education, Federal University of Rio de Janeiro, Rio de Janeiro, Brazil; 2 Institute of Neurology Deolindo Couto, Federal University of Rio de Janeiro, Rio de Janeiro, Brazil; 3 Graduate Program in Neurology and Neuroscience, Fluminense Federal University, Niterói, Rio de Janeiro Brazil

**Keywords:** COVID-19, Parkinson’s disease, Telehealth, Telerehabilitation

Since COVID-19 was declared a pandemic, many countries have enforced a strict shelter-in-place policy to contain the spread of the virus [1]. There is little consensus in the literature over how health workers should proceed concerning patients with chronic neurological diseases, including people with Parkinson’s (PwP) during the COVID-19 pandemic. These patients require long-term care to decrease the severity of the disease amid the pandemic. Therefore, we will propose strategies that may be employed by health workers who care for PwP during the COVID-19 pandemic and may be adapted to suit different social and clinical circumstances (Fig. 1).

**Fig. 1:**
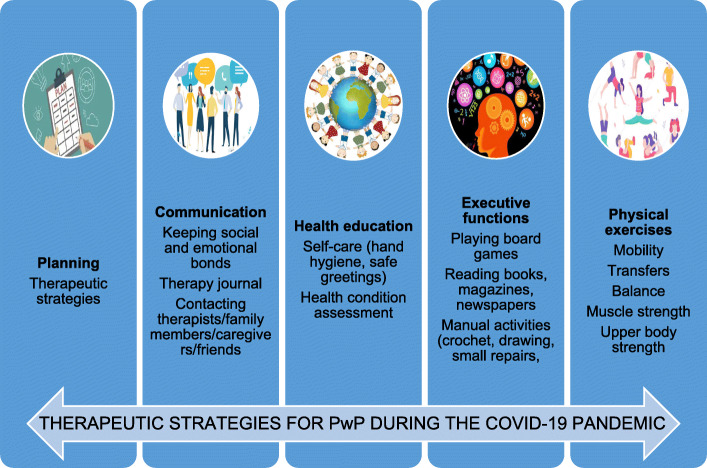
Therapeutic strategies for people with Parkinson’s during the COVID-19 pandemic

Many countries have enforced stay-at-home orders, quarantine, or lockdown; consequently, health workers are unable to care for PwP in person. Thus, it is important for health workers to verify whether there are resolutions and/or regulations of their professional associations that allow teleconsultation or telemonitoring of PwP. If so, some factors should be considered when caring for PwP.

Once a health worker sets a therapeutic plan, the next stage is to complete the activities. It is worth mentioning that planning is a continuous activity because health workers must continually and systematically assess whether the strategies are meeting the therapeutic goals set by the plan.

Health workers must encourage communication between PwP and their families, caregivers, and friends to minimize the effects of social distancing and isolation. One strategy to monitor the activities of PwP is to adopt a therapy journal, in which health workers ask PwP to write on a page the date, time, and which activities were performed throughout the day. Health workers may promote and encourage social and emotional bonds through video calls, such as through WhatsApp. However, currently, WhatsApp has a limit on the number of participants of a given videoconference call. However, many PwP find it difficult to handle new technologies, which should be considered by the health professionals responsible for them.

The education on health for PwP, families, and caregivers during the COVID-19 pandemic cannot be understated. Health workers must provide information on self-care (handwashing, safe greetings) to minimize SARS-CoV-2 contamination. Additionally, health workers can hand the World Health Organization’s communication materials about COVID-19 to PwP. A unique experience of our research group is that during the COVID-19 pandemic, we designed an online form for the awareness of the health conditions of PwP based on the Freezing of Gait Questionnaire (FOG-Q) and Parkinson’s Disease Questionnaire-39 (PDQ-39). Thus, PwP are able to get their FOG-Q results and total PDQ-39 score and their domains via email. This form is free of charge to all Portuguese-speaking PwP (form available at https://bit.ly/2WWGfhX).

Health workers should also recommend activities that involve executive functions suggesting several activities for PwP that involve executive functions, such as reading, listening to music, and singing. It is also important for health workers to be aware of the impacts of negative news regarding COVID-19 on the mental health of PwP. There may be an increase in anxiety and/or depression, which may negatively impact the clinical picture. One way to minimize anxiety and depression symptoms is to advise PwP to avoid watching the news. During the COVID-19 pandemic, health workers must offer support to PwP through telemonitoring or teleconsultations. Although telehealth programs have been used in rehabilitation for many years, the implementation of telehealth programs to service PwP remain in its infancy [2].

Physical exercises are excellent strategies for the management of PwP, resulting in significant benefits [3], which are fundamental to PwP during the COVID-19 pandemic. There are apps with specific exercises based on the main symptomatic treatments for Parkinson’s disease that physiotherapists, occupational therapists, and physical educators can recommend to PwP during teleconsultation and telemonitoring sessions [4–6]. Patients should be advised to practice only those exercises recommended by health professionals. With the large-scale exposure of free workouts through social media, PwP can select unsuitable physical exercises that can be hazardous, resulting in falls or fractures.

When prescribing physical exercises, we should also consider that Parkinson’s may compromise balance in moderate and advanced stages of the disease [7]. PwP patients are a high fall risk with moderate and advanced exercises at home. Fallings place the patient at risk for hospitalization that carries the risk of SARS-COV-2 transmission. Whenever possible, the presence of a caregiver decreases the risk of falls and, consequently, allows for more variations in therapy prescribed to PwP [8, 9].

It is imperative that health workers change their practices and behaviors in order to ensure the physical, psychological, and social well-being of PwP during the COVID-19 pandemic in order to avoid the aggravation of Parkinson’s. Further research remains needed to fully establish the pros and cons of telehealth programs for PwP.

## Data Availability

Not applicable.

## Abbreviations

COVID-19: Coronavirus disease 2019
FOG-Q: Freezing of Gait Questionnaire
PDQ-39: Parkinson’s Disease Questionnaire
PwP: People with Parkinson’s
SARS-CoV-2: Severe acute respiratory syndrome coronavirus 2

## References

[1] Pereira A (2020). Long-term neurological threats of COVID-19: a call to update the thinking about the outcomes of the coronavirus pandemic. Front Neurol..

[2] Quinn L, Macpherson C, Long K, Shah H. Promoting physical activity via Telehealth in people with Parkinson disease: the path forward after the COVID-19 pandemic? Phys Ther:pzaa128. 10.1093/ptj/pzaa128. 10.1093/ptj/pzaa128 PMC7454884 32734298

[3] Schenkman M, Moore CG, Kohrt WM (2018). Effect of high-intensity treadmill exercise on motor symptoms in patients with de novo Parkinson disease. JAMA Neurol..

[4] Block VAJ, Pitsch E, Tahir P, Cree BAC, Allen DD, Gelfand JM (2016). Remote physical activity monitoring in neurological disease: a systematic review. PLoS ONE..

[5] Seidler KJ, Duncan RP, McNeely ME, Hackney ME, Earhart GM (2017). Feasibility and preliminary efficacy of a telerehabilitation approach to group adapted tango instruction for people with Parkinson disease. J Telemed Telecare..

[6] van der Kolk NM, de Vries NM, Kessels RPC (2019). Effectiveness of home-based and remotely supervised aerobic exercise in Parkinson’s disease: a double-blind, randomised controlled trial. Lancet Neurol..

[7] Evans JR, Mason SL, Williams-Gray CH (2011). The natural history of treated Parkinson’s disease in an incident, community based cohort. J Neurol Neurosurg Psychiatry..

[8] Cikajloa I, Hukić A, Dolinšeka I (2018). Can telerehabilitation games lead to functional improvement of upper extremities in individuals with Parkinson’s disease?. Int J Rehabil Res..

[9] Gandolfi M, Geroin C, Dimitrova E (2017). Virtual reality telerehabilitation for postural instability in Parkinson’s disease: a multicenter, single-blind, randomized, controlled trial. BioMed Res Int..

